# News and Coming Events

**Published:** 1908-11-28

**Authors:** 


### News and Coming Events.

Dr. R. W. Allen has been appointed Clinical Pathologist, and Mr. G. Seccombe Hett, Surgeon Laryngologist to the Mount Vernon Hospital for Consumption and Diseases of the Chest.

The Drapers' Company have granted £500, to be paid in five annual instalments of £100, to the Middlesex Hospital Cancer Research Fund to assist the governors in maintaining the investigations which are being pursued into the cause of cancer and its cure.

The Therapeutical and Pharmacological Section of the Royal Society of Medicine will meet on Tuesday, December 1, at 4.30 p.m., at 20 Hanover Square, W. Professor Ralph Stockman will read a paper on "The Action of Salicylates in Rheumatic Affections"; and Dr. David B. Lees will read one on "The Effective Treatment of Acute and Subacute Rheumatism."

The annual dinner of the Royal Free Hospital and School of Medicine for Women will be held at the Hôtel Cecil on Wednesday, December 9, at 6.45 for 7.15 p.m. Miss Aldrich Blake, M.D., M.S., will take the chair. There will be music and a conversazione after dinner. Application for dinner-tickets should be sent to the Honorary Secretaries—Mrs. Florence E. Willey and Mr. Geo. P. Mudge—not later than Saturday, November 28.

On November 23 a very enjoyable concert was provided for the in-patients of the Cancer Hospital (Free) Fulham Road, London, by Madame Mabel Marsden (granddaughter of the founder of the institution and daughter of the late Dr. Alexander Marsden, late Senior Surgeon), assisted by Madame Emilie Steed, the Misses Fanny Woolf, Phillis Bateman, Frieda Johnson, Messrs. Leo Pargeter, Irvin Bromley, Richard Edwyn, and Harry Briden. The programme, which comprised pianoforte and violin solos, songs, and recitations, was excellently carried out and much enjoyed.

The twenty-fourth annual meeting of Fellows and Members of the Royal College of Surgeons was held on November 19, and was largely attended. Mr. Joseph Smith moved a resolution thanking the Council for having taken a poll of the members on the question of the admission of women to the College diplomas, but expressing regret that the Council found itself unable to abide by the result, and trusting that the Council would shortly take a further poll of the Fellows and Members on the question of direct representation of members on the College Council. The resolution was carried almost unanimously. Dr. W. G. Dickinson moved that "This meeting is of opinion that women, when admitted to the diplomas of the College, should have equal rights with men." This was also carried. In reply to a member of the College who asked that steps should be taken to ensure that improper use be not made of deceased members' diplomas, the Chairman stated that a printed notice is now being affixed to the diplomas issued by the College asking that on the death of the diplomate the diploma should be returned to the College. Mr. F. W. Collingwood moved a resolution asking the Council "to use their moral influence to obtain some more sufficient title for those who have passed the examinations for the conjoint diploma, remembering the importance attached to degrees to the detriment of holders of the said diploma granted by this ancient corporation in conjunction with the Royal College of Physicians of London." This was carried, and a vote of thanks to the President, Mr. Henry Morris, for presiding closed the proceedings.

Mr. Sydney R. Scott, M.S.Lond., F.R.C.S.Eng., has been appointed Assistant Aural Surgeon to St. Bartholomew's Hospital.

Dr. T. G. Brodie, M.D., F.R.S., has resigned the Professor-Superintendentship of the Brown Animal Sanitary Institution, in connection with the University of London, upon his appointment to the Chair of Physiology at the University of Toronto.

The Seamen's Hospital Society has received from the Government of France a contribution of 500 francs. The Government of Spain and the Government of the Commonwealth of Australia have also become annual contributors to the Society of £10 and £25 respectively.

During the summer a deputation, formed by the Hon. Secretary, Dr. Arthur Haydon, from the Brussels Medical Graduates' Association, had an interview with Professor Rommelaere, the Rector of the University of Brussels. We are informed that on the representations of the Brussels Medical Graduates' Association the following concessions were promised to candidates for the M.D. degree:—(1) That it should be sufficient for English candidates to produce their certificates of registration instead of their diplomas on presenting themselves for examination at Brussels. (2) That the attention of the Administrative Council of the University should be drawn to the insufficiency of certain Indian and American qualifications which do not entitle their holders to the full privilege of practice in their own country. (3) That the examination for the M.D. degree in February should in future take place in March, this month being more convenient for English candidates.

### GENERAL MEDICAL COUNCIL.

The eighty-eighth session of the General Medical Council was commenced on November 24, Sir Donald Macalister, the President, being in the chair. The President, in his opening address, said that the Council that day entered upon its second half century. The Council had at first to contend with grave difficulties, internal and external, but it had kept before it its original purpose—the safeguarding of the public health and well-being—and it had promoted in a remarkable degree the improvement of the profession of medicine. On the work it had succeeded in accomplishing, notwithstanding its restrictions, it might well rest its claim to be entrusted with better equipment and a freer field of action. The President referred with pleasure to the appointment by the University of Cambridge of Sir Clifford Allbutt to the seat which he himself had had the honour to occupy from 1889 onwards. Several questions of national importance had been submitted for their consideration by the Lord President of the Privy Council. The Bill which had been proposed for the purpose of obviating some of the dangers arising from the administration of anæsthetics by untrained or unqualified persons contained certain provisions which appeared, to him at least, to be questionable, and on these points he had, on behalf of the Council, been in communication with the authorities. The President stated that by the assistance of the Colonial Office and of the Foreign Office a large mass of authoritative information had been obtained respecting the laws regulating the practice of medicine, and the restrictions imposed on unqualified practitioners throughout the Empire and in foreign countries. This information had been carefully analysed and digested, and the publication of the digest was contemplated.

